# Antihypertensive medication use during pregnancy in a real-world cohort of patients diagnosed with a hypertensive disorder of pregnancy

**DOI:** 10.3389/fcvm.2023.1225251

**Published:** 2023-07-07

**Authors:** Julian E. Garcia, Ian R. Mulrenin, Anh B. Nguyen, Matthew S. Loop, Melissa A. Daubert, Rachel Urrutia, Craig R. Lee

**Affiliations:** ^1^ Division of Pharmacotherapy and Experimental Therapeutics, UNC Eshelman School of Pharmacy, University of North Carolina at Chapel Hill, Chapel Hill, NC, United States; ^2^ Department of Health Outcomes Research and Policy, Harrison College of Pharmacy, Auburn University, Auburn, AL, United States; ^3^ Division of Cardiology, Duke University School of Medicine, Duke Clinical Research Institute, Duke University, Durham, NC, United States; ^4^ Division of General Obstetrics and Gynecology, Department of Obstetrics and Gynecology, UNC School of Medicine, University of North Carolina at Chapel Hill, Chapel Hill, NC, United States

**Keywords:** hypertensive disorders of pregnancy, preeclampsia, gestational hypertension, antihypertensive medication, labetalol, nifedipine, hydralazine, methyldopa

## Abstract

Hypertensive disorders of pregnancy (HDP) are rising in prevalence and associated with adverse maternal and infant health outcomes. Current guidelines recommend labetalol, nifedipine, and methyldopa as acceptable first-line agents to treat HDP in outpatient settings. However, the current practice regarding antihypertensive medication usage and selection remain unclear. A retrospective, observational cohort study was conducted in 1,641 patients with a physician diagnosis of HDP who delivered at two academic medical centers in North Carolina from 2014 to 2017. Use of any antihypertensive medication, and the agent selected, at any encounter during pregnancy or on the delivery date was collected from the electronic health record. Proportions were compared across HDP diagnosis (eclampsia/severe preeclampsia, chronic hypertension with superimposed preeclampsia, preeclampsia, gestational hypertension) by Chi-square tests and multivariable logistic regression. Antihypertensive medications were used in 1,276 (77.8%) patients overall. Among treated patients, labetalol (74.9%) was the most frequently used medication followed by nifedipine (29.6%) and hydralazine (20.5%). Methyldopa was used infrequently (4.4%). HDP type was the strongest factor associated with use of an antihypertensive agent. Relative to gestational hypertension, antihypertensive use was significantly more likely [odds ratio (95% CI)] in patients with severe preeclampsia [5.94 (3.85–9.16)], chronic hypertension with superimposed preeclampsia [4.99 (3.46–7.19)], and preeclampsia [2.13 (1.61–2.82)]. In a real-world setting, antihypertensive medication use among HDP patients was common, labetalol, nifedipine, and hydralazine were the most commonly selected agents, and increasing HDP severity was associated with a higher likelihood of antihypertensive use. Future studies comparing medication effectiveness in pregnant patients with distinct HDP diagnoses are needed.

## Introduction

Hypertensive disorders of pregnancy (HDP) are among the most common disorders in pregnant people, affecting approximately 10% of pregnancies worldwide (1). Due to delayed childbearing, and the global obesity epidemic, the prevalence of HDP increased by 80% between 1995 and 2008 and continues to rise (2–4). HDP are comprised of three distinct clinical presentations: gestational hypertension (new onset hypertension without proteinuria), preeclampsia (new onset hypertension with proteinuria and with or without severe features of end-organ dysfunction), and chronic hypertension with superimposed preeclampsia (hypertension prior to pregnancy with preeclampsia features) (5, 6). Hypertension in pregnancy is defined as a blood pressure (BP) ≥140/90 mmHg on at least two occasions 4 h apart and severe range hypertension is defined as BP ≥160/100 mmHg that is confirmed as persistent with a second reading in 15 min (5, 6).

HDP is associated with an increased risk of fetal morbidity and mortality, as well as maternal sequelae including stroke, pulmonary edema, and death (2–7). Therefore, antihypertensive medications are often used to lower BP, prolong the pregnancy, and improve maternal and neonatal health outcomes (7). Current guidelines recommend use of antihypertensive medications during pregnancy for the treatment of severe BP elevations and chronic hypertension; labetalol, nifedipine, and methyldopa are designated as acceptable first-line, chronically administered agents to treat HDP, and intravenous hydralazine, intravenous labetalol, and immediate-release nifedipine are recommended as acceptable initial agents for severe hypertension (1, 8, 9). Second-line agents include hydrochlorothiazide, furosemide, nicardipine, and clonidine (5, 10). Due to the various physiologic changes experienced during pregnancy, pregnancy-associated changes in pharmacokinetics and pharmacodynamics of many drugs, including antihypertensive medications, occur in pregnant individuals (11). Accordingly, the best practices for when to initiate medications, which medication to select as initial therapy, and how to optimally dose each medication remain unclear due to a lack of clinical evidence comparing the safety and effectiveness of different treatment strategies.

Various barriers and logistical challenges have limited the recruitment of pregnant patients into clinical trials, which has limited the amount of data regarding the efficacy and safety of drugs in pregnancy (12). This lack of data has limited the strength and specificity of guideline recommendations for HDP treatment, which are largely based on expert opinion and/or small studies, retrospective analyses, or observational registries (9). Consequently, medications used to treat HDP may be ordered for pregnant individuals off-label and via trial-and-error approaches. However, the degree of interpatient differences in medication selection and dosing for HDP in clinical practice, factors associated with these prescribing practices, and the impact on BP control and clinical outcomes remain poorly understood (10). Studies that characterize antihypertensive medication use and selection during pregnancy in diverse real-world clinical settings are needed. Therefore, the objectives of this study were to (1) describe the frequency of antihypertensive medication use and agent selection in a large real-world cohort of patients diagnosed with HDP, (2) compare the frequency of antihypertensive use and selection of specific medications across patient groups with distinct HDP diagnoses, and (3) identify demographic and clinical factors associated with the use and selection of antihypertensive medications.

## Materials and methods

### Study population

This retrospective, observational cohort study utilized a previously assembled dataset consisting of pregnant individuals with new onset HDP who delivered at the University of North Carolina at Chapel Hill (UNC-Chapel Hill) or Duke University between 2007 and 2017 (Supplementary Figure S1). Women with an International Classification of Disease (ICD) 9 or 10 diagnosis code for HDP from an inpatient or outpatient encounter were included (*N* = 9,782). In women with multiple pregnancies complicated with HDP, the first pregnancy served as the index pregnancy. Individuals with an HDP diagnosis greater than 6 months prior to or 6 weeks after delivery date (*N* = 552), an invalid gestational age or delivery before Epic electronic health records (EHR) implementation at each site in 2014 (*N* = 3,623), and no medication data available during pregnancy or on the date of delivery (*N* = 3,966) were excluded. The remaining 1,641 patients were included in the final dataset for analysis. This study was approved by the institutional review boards at Duke University and UNC-Chapel Hill.

### Data collection

Demographic characteristics collected from the EHR included race, ethnicity, age, insurance status, site of delivery, and year of delivery. Significant past medical history (history of hypertension, hyperlipidemia, diabetes mellitus, and renal disease) and pregnancy characteristics (gestational age of onset of HDP, gestational diabetes) were also collected. Race and ethnicity were based on self-report as recorded in the EHR. Race was classified as White, Black, or Other (defined as Pacific Islander, Native American, or unknown race), and ethnicity was classified as Hispanic or Not Hispanic. HDP type was classified based on diagnoses codes from ICD-9 (642.5: severe preeclampsia, 642.7: preeclampsia or eclampsia superimposed on pre-existing hypertension) and ICD-10 (O11: pre-existing hypertension with preeclampsia, O13: gestational hypertension without significant proteinuria, O14: preeclampsia, O15: eclampsia). HDPs were ranked in order of increasing severity: gestational hypertension, preeclampsia, chronic hypertension with superimposed preeclampsia, and severe preeclampsia including eclampsia.

The primary endpoint was treatment with an antihypertensive medication (yes/no) at any inpatient or outpatient encounter during pregnancy and/or on the date of delivery. Among the patients that received treatment, medication class (beta blocker, calcium channel blocker, vasodilator, alpha agonist, diuretic) was a co-primary endpoint. Specific medications within the medication classes (e.g., labetalol, nifedipine, hydralazine, methyldopa, hydrochlorothiazide) and the number of antihypertensive medication classes used (one, two, three or more) were secondary endpoints.

### Data analysis

Baseline characteristics and the frequency of the primary endpoints were described in the overall study population, and compared across HDP type (eclampsia/severe preeclampsia, chronic hypertension with superimposed preeclampsia, preeclampsia, and gestational hypertension), by descriptive statistics. An analysis conducted exclusively within the subset of 1,276 patients treated with an antihypertensive medication compared the medication class and individual medication endpoints across HDP type. A Chi-square test was used to determine whether differences in the proportion of the primary and secondary endpoints existed across HDP diagnosis categories. To account for implementation of ICD-10 codes for diagnosing HDP in October 2015, a sensitivity analysis describing the frequency of antihypertensive use and selection across HDP type was conducted solely within patients who delivered in 2016 and 2017.

In order to identify demographic and clinical factors associated with the use and selection of antihypertensive medications, demographic and clinical factors were compared across those treated versus not treated and those treated versus not treated with the most commonly ordered classes of agents (beta blockers, calcium channel blockers, vasodilators). We evaluated whether differences in each factor existed across medication groups (yes/no) using Chi-square or Student's *t*-test, as appropriate, and determined the magnitude of the associations utilizing logistic regression. Unadjusted odds ratios (ORs) and 95% confidence intervals (95% CI) were calculated to determine whether each demographic and clinical factor was associated with each medication endpoint [treatment with any antihypertensive, medication classes used (beta blocker, calcium channel blocker, vasodilator)]. To determine which factors were independently associated with antihypertensive use (yes/no), beta blocker use (yes/no), calcium channel blocker use (yes/no), and vasodilator use (yes/no), adjusted ORs and 95% CIs were also calculated for each demographic and clinical factor in a multivariate logistic regression model for each endpoint. The data for this cohort was analyzed with SAS-JMP Pro v15.2. *P*-values <0.05 were considered statistically significant.

## Results

### Demographic and clinical characteristics of the total study population

The demographic and clinical characteristics of the study population (*N* = 1,641) are described in Table 1. The mean (±SD) age of the population was 30.5 ± 6.5 years, and 41.0% patients self-identified as Black race and 15.7% patients self-identified as Hispanic. In addition, 30.5% of patients had a history of hypertension, defined as chronic hypertension within 12 months of delivery. The distribution of HDP type was 289 (17.6%) patients with eclampsia/severe preeclampsia, 557 (33.9%) with preeclampsia, 401 (24.4%) with chronic hypertension with superimposed preeclampsia, and 394 (24.0%) with gestational hypertension.

**Table 1:** Demographic and clinical factors in total study population compared across HDP type.

Characteristics	All (*N* = 1,641)	Eclampsia/severe preeclampsia (*N* = 289)	Chronic hypertension with superimposed preeclampsia (*N* = 401)	Preeclampsia (*N* = 557)	Gestational hypertension (*N* = 394)	*P* value
**Age (years)**	30.5 ± 6.5	29.3 ± 6.7	32.2 ± 6.6	29.6 ± 6.4	30.7 ± 6.0	<0.001
**Race**						<0.001
White	604 (36.8%)	110 (38.1%)	129 (32.2%)	193 (34.7%)	172 (43.7%)	** **
Black	673 (41.0%)	107 (37.0%)	196 (48.9%)	216 (38.8%)	154 (39.1%)	** **
Other	364 (22.2%)	72 (24.9%)	76 (19.0%)	148 (26.6%)	68 (17.3%)	** **
**Ethnicity**	** **	** **	** **	** **	** **	<0.001
Hispanic	257 (15.7%)	54 (18.7%)	47 (11.7%)	113 (20.3%)	43 (10.9%)	
Not hispanic or unknown	1,384 (84.3%)	235 (81.3%)	354 (88.3%)	444 (79.7%)	351 (89.1%)	
**Year of delivery**	** **	** **	** **	** **	** **	<0.001
2014–2015	562 (34.3%)	256 (88.6%)	99 (24.7%)	83 (14.9%)	124 (31.5%)	** **
2016–2017	1,079 (65.8%)	33 (11.4%)	302 (75.3%)	474 (85.1%)	270 (68.5%)	** **
**Site of delivery**	** **	** **	** **	** **	** **	0.171
Site A	783 (47.7%)	137 (47.4%)	173 (43.1%)	279 (50.1%)	194 (49.2%)	** **
Site B	858 (52.3%)	152 (52.6%)	228 (56.9%)	278 (49.9%)	200 (50.8%)	** **
**Primary payor**	** **	** **	** **	** **	** **	<0.001
Government	851 (51.2%)	165 (57.1%)	200 (49.9%)	307 (55.1%)	179 (45.4%)	** **
Commercial	630 (38.4%)	116 (40.1%)	154 (38.4%)	191 (34.3%)	169 (42.9%)	** **
Other (including self-pay)	160 (9.8%)	8 (2.8%)	47 (11.7%)	59 (10.6%)	46 (11.7%)	** **
**Pregnancy characteristics**						
Gestational onset of HDP (weeks)	34.8 ± 4.7	31.6 ± 6.2	34.3 ± 4.4	35.0 ± 3.9	36.0 ± 5.1	<0.001
Gestational diabetes	381 (23.2%)	51 (17.7%)	107 (26.7%)	128 (23.0%)	95 (24.1%)	0.043
**Past medical history**						
Hypertension	501 (30.5%)	77 (26.6%)	253 (63.1%)	78 (14.0%)	93 (23.6%)	<0.001
Type 1 DM	77 (4.7%)	12 (4.2%)	26 (6.5%)	29 (5.2%)	10 (2.5%)	0.047
Type 2 DM	191 (11.6%)	18 (6.2%)	69 (17.2%)	56 (10.1%)	48 (12.2%)	<0.001
Prediabetes	84 (5.1%)	20 (6.9%)	18 (4.5%)	23 (4.1%)	23 (5.8%)	0.297
Renal disease	34 (2.1%)	4 (1.4%)	19 (4.7%)	6 (1.1%)	5 (1.3%)	0.001

Various differences in the demographic and clinical characteristics were observed across HDP type (Table 1). Individuals diagnosed with eclampsia/severe preeclampsia were diagnosed with HDP earlier in the pregnancy, were younger in age, and had a lower prevalence of diabetes compared to other HDP types. Patients diagnosed with chronic hypertension with superimposed preeclampsia were older, and contained a higher proportion of Black patients, patients with a known history of hypertension, and patients with a history of renal disease.

### Frequency of antihypertensive medication use and selection

Of the total study population, 1,276 (77.8%) were treated with one or more antihypertensive medications during pregnancy or on the date of delivery. Table 2 describes the frequency of medication classes, specific medication within each class, and the number of distinct antihypertensive medications classes used across HDP type. Beta blockers were the most frequently used medication class (79.2%) and labetalol was the most frequently ordered medication (74.9%) overall. Calcium channel blockers were the second most frequently used medication class (31.8%) and nifedipine was the most commonly ordered medication in this class (29.6%). Vasodilators were the third most frequently used antihypertensive class (21.6%) with hydralazine as the most commonly ordered agent (20.5%). Alpha agonists (5.5%) and diuretics (6.7%) were used at lower frequencies, with methyldopa (4.4%), furosemide (3.5%), and hydrochlorothiazide (2.5%) as the most commonly ordered agents within these classes. Overall, the majority of patients (839, 65.8%) were treated with a single medication class; however, 322 (25.2%) patients were treated with two and 115 (9.0%) patients were treated with three or more distinct medication classes, respectively.

**Table 2:** Antihypertensive medication classes and agents selected across HDP type.

Medication class agent	All (*N* = 1,276)	Eclampsia/severe preeclampsia (*N* = 260)	Chronic with superimposed preeclampsia (*N* = 354)	Preeclampsia (*N* = 425)	Gestational hypertension (*N* = 237)	*P* value
**Beta blockers**	1,011 (79.2%)	222 (85.4%)	299 (84.5%)	329 (77.4%)	161 (67.9%)	<0.001
Labetalol	956 (74.9%)	213 (81.9%)	289 (81.6%)	309 (72.7%)	145 (61.2%)	<0.001
Metoprolol	46 (3.6%)	8 (3.1%)	14 (4.0%)	15 (3.5%)	9 (3.8%)	0.946
Propranolol	26 (2.0%)	6 (2.3%)	6 (1.7%)	6 (1.4%)	8 (3.4%)	0.388
**Calcium channel blockers**	406 (31.8%)	57 (21.9%)	158 (44.6%)	126 (29.7%)	65 (27.4%)	<0.001
Nifedipine	378 (29.6%)	54 (20.8%)	142 (40.1%)	123 (28.9%)	59 (24.9%)	<0.001
Amlodipine	23 (1.8%)	3 (1.2%)	12 (3.4%)	3 (0.71%)	5 (2.1%)	0.035
Diltiazem	11 (0.86%)	2 (0.77%)	8 (2.3%)	0 (0.0%)	1 (0.42%)	0.003
**Diuretics**	86 (6.7%)	15 (5.8%)	33 (9.3%)	25 (5.9%)	13 (5.5%)	0.176
Furosemide	45 (3.5%)	12 (4.6%)	13 (3.7%)	13 (3.1%)	7 (3.0%)	0.711
HCTZ	32 (2.5%)	3 (1.2%)	17 (4.8%)	6 (1.4%)	6 (2.5%)	0.012
**Vasodilators**	275 (21.6%)	88 (33.9%)	83 (23.5%)	85 (20.0%)	19 (8.0%)	<0.001
Hydralazine	261 (20.5%)	84 (32.3%)	80 (22.6%)	83 (19.5%)	14 (5.9%)	<0.001
Nitroglycerin	17 (1.3%)	5 (1.9%)	3 (0.85%)	3 (0.71%)	6 (2.5%)	0.178
**Alpha-Agonist**	70 (5.5%)	10 (3.9%)	31 (8.8%)	9 (2.1%)	20 (8.4%)	<0.001
Methyldopa	56 (4.4%)	7 (2.7%)	28 (7.9%)	5 (1.2%)	16 (6.8%)	<0.001
Clonidine	16 (1.3%)	3 (1.2%)	5 (1.4%)	4 (0.94%)	4 (1.7%)	0.854
**Number of antihypertensive medication classes used**						<0.001
One	839 (65.8%)	156 (60.0%)	185 (52.3%)	298 (70.1%)	200 (84.4%)	
Two	322 (25.2%)	81 (31.2%)	102 (28.8%)	105 (24.7%)	34 (14.3%)	
Three or more	115 (9.0%)	23 (8.8%)	67 (18.9%)	22 (5.2%)	3 (1.3%)	

### Antihypertensive medication use and selection across HDP type

**Figure 1:**
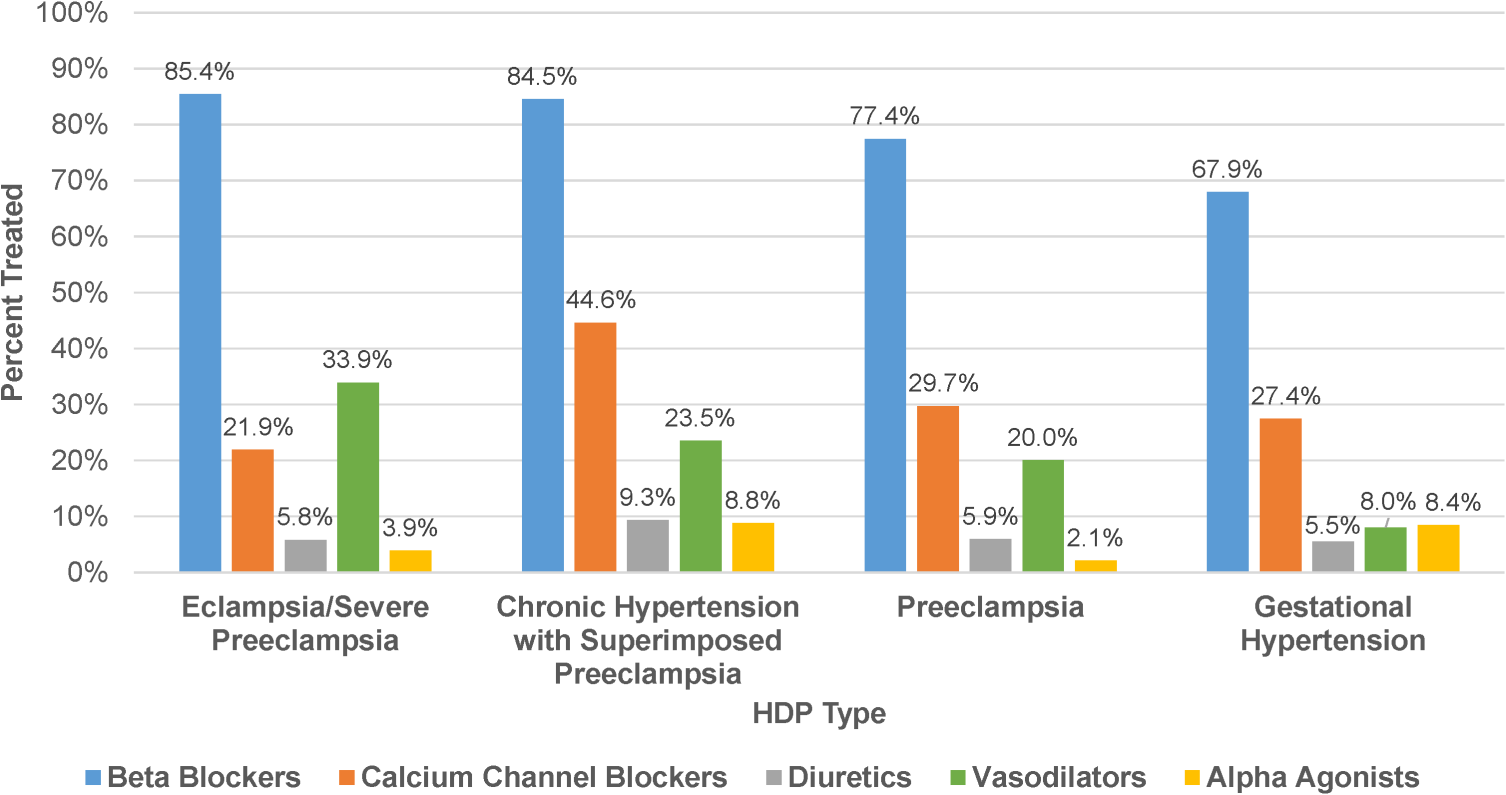
Antihypertensive medication use by class. Frequency of antihypertensive medication classes used among patients treated for HDP (*N* = 1,276) stratified by HDP type.

The percentage of patients treated with an antihypertensive medication significantly differed across descending severity of HDP type (*P* < 0.001): eclampsia/severe preeclampsia, (90.0%); chronic hypertension with superimposed preeclampsia, (88.3%); preeclampsia, (76.3%); and gestational hypertension, (60.2%). Among patients treated with an antihypertensive, beta blockers were the most frequently ordered medication class in each HDP type (67.9%–85.4%, Figure 1). Calcium channel blockers were more frequently ordered in chronic hypertension with superimposed preeclampsia (44.6%) compared to other HDP types (21.9%–29.7%), while vasodilators were more frequently ordered in eclampsia/severe preeclampsia (33.9%) compared to other HDP types (8.0%–23.5%). Although infrequently ordered overall, alpha agonists were used more commonly in chronic hypertension with superimposed preeclampsia and gestational hypertension (8.4%–8.8%) compared to other HDP types (2.1%–3.9%). As summarized in Table 2, there also was a significant difference in the proportion of patients treated with multiple antihypertensive medication classes across HDP type (*P* < 0.001). Among treated patients, patients diagnosed with eclampsia/severe preeclampsia (40.0%) and chronic hypertension with superimposed preeclampsia (47.7%) were more likely to be treated with two or more medication classes than those diagnosed with preeclampsia (29.9%) and gestational hypertension (15.6%). The sensitivity analysis evaluating medication use and selection in those who delivered in 2016–2017 demonstrated results similar to the primary analysis (Supplementary Figure S2).

### Demographic and clinical factors associated with the use and selection of antihypertensive medications

A comparison of demographic and clinical factors across patients who received and did not receive treatment with an antihypertensive drug is summarized in Table 3. Multivariate logistic regression analysis demonstrated that several factors were associated with the use of an antihypertensive medication. HDP type was the strongest independent predictor of treatment. Relative to patients diagnosed with gestational hypertension, antihypertensive medication use was significantly higher in patients diagnosed with eclampsia/severe preeclampsia (adjusted OR: 6.66; 95% CI: 3.25–13.66), chronic hypertension with superimposed preeclampsia (adjusted OR: 4.51; 95% CI: 2.91–6.98), or preeclampsia (adjusted OR: 2.70; 95% CI: 1.94–3.75). Other factors associated with higher prevalence of antihypertensive medication use included a known history of hypertension (adjusted OR: 2.11; 95% CI: 1.45–3.06) and having commercial insurance relative to government insurance coverage (adjusted OR: 1.81; 95% CI: 1.30–2.53). Factors associated with lower prevalence of medication use included later gestational age of onset of HDP and diagnosis of gestational diabetes, type 1 diabetes mellitus, or type 2 diabetes mellitus. Maternal age, race, and ethnicity did not have statistically significant associations with treatment with an antihypertensive medication.

**Table 3:** Demographic and clinical factors across patients treated (Tx) with an antihypertensive medication.

Characteristics	Tx yes (*N* = 1,276)	Tx no (*N* = 365)	Unadjusted OR	95% CI	*P* value	Adjusted OR^b^	95% CI	*P* value
Age (years)	30.4 ± 6.6	30.8 ± 6.0	0.99	0.97, 1.01	0.280	0.99	0.96, 1.01	0.265
Black race	534 (41.9%)	139 (38.1%)	1.17	0.92, 1.49	0.197	1.05	0.77, 1.45	0.744
Hispanic ethnicity	185 (14.5%)	72 (19.7%)	0.69	0.51, 0.93	0.016	0.89	0.58, 1.35	0.575
Year of delivery								
2014–2015	463 (36.3%)	99 (27.1%)	Reference			Reference		
2016–2017	813 (63.7%)	266 (72.9%)	0.65	0.51, 0.85	0.001	1.11	0.74, 1.65	0.613
Site of delivery								
Site A	591 (46.3%)	192 (52.6%)	Reference			Reference		
Site B	685 (53.7%)	173 (47.4%)	1.29	1.02, 1.62	0.034	1.33	0.98, 1.80	0.066
Primary payor								
Government	640 (50.2%)	211 (57.8%)	Reference			Reference		
Commercial	515 (40.4%)	115 (31.5%)	1.48	1.14, 1.91	0.003	1.81	1.30, 2.53	0.001
Other (including self-pay)	121 (9.5%)	39 (10.7%)	1.02	0.69, 1.52	0.910	1.38	0.85, 2.23	0.190
Hypertensive disorders of pregnancy (HDP)								
Eclampsia/severe preeclampsia	260 (20.4%)	29 (8.0%)	5.94	3.85, 9.16	<0.001	6.66	3.25,13.66	<0.001
Chronic hypertension with superimposed preeclampsia	354 (27.7%)	47 (12.9%)	4.99	3.46, 7.19	<0.001	4.51	2.91, 6.98	<0.001
Preeclampsia	425 (33.3%)	132 (36.2%)	2.13	1.61, 2.82	<0.001	2.70	1.94, 3.75	<0.001
Gestational hypertension	237 (18.6%)	157 (43.0%)	Reference			Reference		
Pregnancy characteristics								
Gestational onset of HDP (weeks)^a^	34.4 ± 4.7	35.8 ± 4.7	0.93	0.90, 0.96	<0.001	0.96	0.93, 1.00	0.031
Gestational diabetes	219 (17.2%)	162 (44.4%)	0.26	0.20, 0.33	<0.001	0.35	0.25, 0.49	<0.001
Past medical history								
Hypertension	431 (33.8%)	70 (19.2%)	2.15	1.62, 2.86	<0.001	2.11	1.45, 3.06	<0.001
Type 1 DM	38 (3.0%)	39 (10.7%)	0.26	0.16, 0.41	<0.001	0.36	0.19, 0.67	0.001
Type 2 DM	110 (8.6%)	81 (22.2%)	0.33	0.24, 0.45	<0.001	0.55	0.36, 0.84	0.006
Prediabetes	53 (4.2%)	31 (8.5%)	0.47	0.29, 0.74	0.001	0.58	0.32, 1.04	0.067
Renal disease	27 (2.1%)	7 (1.9%)	1.11	0.48, 2.56	0.815	0.71	0.27, 1.89	0.493

^a^ Data only available in *N* = 1,357.

^b^ Adjusted analysis was conducted in the *N* = 1,357 with gestational onset of HDP data available.

Various factors were also associated with use of specific medication classes. HDP type was the only factor associated with treatment with a beta blocker (Supplementary Table S1), with diagnosis of eclampsia/severe preeclampsia (adjusted OR: 2.00; 95% CI: 1.04–3.82), chronic hypertension with superimposed preeclampsia (adjusted OR: 2.44; 95% CI: 1.50–3.96), and preeclampsia (adjusted OR: 1.55; 95% CI: 1.03–2.34) each associated with more frequent use of a beta blocker relative to diagnosis of gestational hypertension. Treatment with a calcium channel blocker was associated with several factors including Black race, year of delivery, site of delivery, diagnosis of chronic hypertension with superimposed preeclampsia relative to diagnosis of gestational hypertension, earlier gestational age of onset of HDP, and history of renal disease (Supplementary Table S2). Treatment with a vasodilator was associated with Black race, site of delivery, HDP type, and earlier gestational age of onset of HDP (Supplementary Table S3).

## Discussion

In this diverse, real-world cohort of patients diagnosed with HDP at two large academic medical centers in North Carolina, 78% of the total population received treatment with an antihypertensive medication during pregnancy or on the date of delivery. The type of HDP was the strongest independent predictor of treatment with an antihypertensive. Patients diagnosed with eclampsia/severe preeclampsia, chronic hypertension with superimposed preeclampsia, and preeclampsia were significantly more likely to be treated with an antihypertensive compared to the mildest HDP type, gestational hypertension. Known history of hypertension and commercial insurance were also significantly associated with a higher frequency of antihypertensive medication use. In addition to HDP type, patient race, insurance status, and past medical history were factors associated with the decision to treat with certain medication classes. These data suggest that the decision to prescribe an antihypertensive drug and to select a specific antihypertensive drug is complex and is influenced by multiple factors.

Clinical practice guidelines recommend that antihypertensive medications are indicated during pregnancy to lower BP in patients diagnosed with severe hypertension (SBP ≥160 mmHg or DBP ≥110 mmHg) and for the treatment of chronic hypertension (1, 8, 9, 13). A recent American Heart Association (AHA) scientific statement for hypertension in pregnancy acknowledged the controversies that exist in the treatment of HDP including when medication is indicated, BP goals, and selection of antihypertensive medication (10). However, the recent CHAP (Chronic Hypertension and Pregnancy) Trial found that treating women with mild chronic hypertension during pregnancy to a BP target of <140/90 mmHg was associated with better pregnancy outcomes compared to a strategy of reserving treatment only for those with severe hypertension (14). Given the potential benefit of more aggressive treatment of elevated BP in pregnancy, as well as the American College of Obstetricians and Gynecologists (ACOG) now recommending utilizing 140/90 mmHg as the threshold for initiation or titration of medical therapy for chronic hypertension in pregnancy (15), it is likely that there will be greater utilization of antihypertensive medications during pregnancy.

The current study in a real-world cohort of patients diagnosed with HDP found that approximately 8 in 10 pregnant women with HDP received treatment with an antihypertensive medication during pregnancy or on the date of delivery, a high prevalence overall when considering the debate and uncertainty surrounding when antihypertensive medications are clinically indicated in pregnancy. The guidelines recommend pharmacologic treatment for more severe types of HDP, while there is limited guidance for gestational hypertension (8–10). Although a high rate of treatment was observed overall, these rates differed by the HDP type and appear to be consistent with the guideline recommendations. Patients diagnosed with eclampsia/severe preeclampsia, chronic hypertension with superimposed preeclampsia, and preeclampsia, which are more frequently treated in an inpatient setting, each exhibited a significantly higher probability of treatment with an antihypertensive relative to gestational hypertension.

The decision of which antihypertensive to initiate for BP treatment during pregnancy is largely based on clinical judgement on a per-patient basis (8–10). The current study found that among patients prescribed an antihypertensive medication, beta blockers were the most commonly prescribed medication class, and labetalol was the most commonly ordered medication across all HDP types. Labetalol is available in both oral and intravenous formulations, and can be used both chronically for longer term BP control as well as acutely in patients with severe hypertension. In addition, the calcium channel blocker, nifedipine, and the vasodilator, hydralazine, also were frequently used. Current practice guidelines recommend labetalol, nifedipine, and methyldopa as first-line agents for pre-existing hypertension to prevent the progression to severe hypertension in pregnant patients (7–10). Certain guidelines and statements mention that it is reasonable to manage pre-existing chronic hypertension in patients who became pregnant with previously prescribed antihypertensives, provided the agent is not contraindicated in pregnancy (9, 10, 13). For severe hypertension, intravenous labetalol, hydralazine, and immediate-release nifedipine are comparable with respect to safety and efficacy, and guidelines recommend that providers choose based on experience and familiarity with a particular drug (7, 10). The most frequently ordered medications for HDP in this cohort were the beta blocker labetalol, followed by the calcium channel blocker nifedipine, which is consistent with guideline recommendations. Alpha agonists, such as methyldopa, however, were not as frequently ordered despite guideline recommendations. This may in part be the result of the limited or lack of supply of methyldopa in the last decade, as well as the likelihood that a greater proportion of treated patients in this cohort received an antihypertensive in the inpatient rather than the outpatient setting. Following calcium channel blockers, the next most common medication class used was the vasodilators, specifically hydralazine. As expected, there was a notably high prevalence of vasodilator use in the setting of eclampsia/severe preeclampsia. Labetalol and nifedipine were also commonly used in this HDP type. These results are consistent with guideline recommendations and evidence supporting the safety and efficacy of hydralazine, labetalol, and nifedipine for the treatment of severe hypertension during pregnancy. Second-line agents, such as hydrochlorothiazide, furosemide and clonidine, were not frequently used in our cohort.

The number of antihypertensive medication classes used in this cohort varied across HDP type. Overall, the majority of the population received treatment with a single antihypertensive class and approximately one-third of patients received medications from two or more classes. Patients with gestational hypertension, the least severe HDP type, were least likely to receive more than one antihypertensive medication class; however, patients with severe preeclampsia and chronic hypertension with superimposed preeclampsia were commonly treated with multiple medication classes. These results suggest that severity of HDP type also is associated with use of multiple antihypertensive medication classes to control BP.

In addition to HDP type, other factors were associated with medication use and selection. Patients treated at one site had a lower probability of treatment with a calcium channel blocker and a higher probability of treatment with a vasodilator compared to patients treated at the other site. A reason for differences in ordering practices between sites of delivery could be related to individual clinician preference for an agent and the institution's predetermined treatment algorithm. Although patient race was not significantly associated with the decision to treat with an antihypertensive, Black patients had a higher probability of receiving treatment with a calcium channel blocker or with a vasodilator when compared to White patients. Hypertension guidelines outside of pregnancy have recommended calcium channel blockers as a first-line treatment for hypertension in Black patients (8), suggesting that this recommendation may be extrapolated to ordering decisions in the pregnant population. Patients with a past medical history of hypertension had a significantly higher probability of being ordered any antihypertensive medication compared to patients without a known history of hypertension. These observations are consistent with recommendations to manage pre-existing chronic hypertension in patients who became pregnant with previously prescribed antihypertensives, provided the agent is not contraindicated in pregnancy (9, 13). Diabetes was associated with a lower probability of being treated with an antihypertensive agent. This unanticipated observation requires further study, but could be related to use of blood glucose lowering agents and hesitation from prescribers to initiate additional medications.

Recent recommendations from the United States Preventative Services Task Force (USPSTF) for BP monitoring at every prenatal visit for asymptomatic pregnant patients may increase the incidence of HDP diagnoses across the population. This recommendation is based on the ability to effectively identify and treat HDP to prevent the associated morbidity and mortality related to these conditions. Specific guidance on pharmacologic treatment is therefore critically needed (16). Initial selection and dosing strategies are key areas of research for future studies that aim to evaluate the impact of antihypertensive pharmacotherapy on BP control and clinical outcomes in pregnant individuals diagnosed with HDP.

In the CHAP trial, labetalol and extended-release nifedipine were the preferred initial agents used for BP lowering in the setting of chronic hypertension during pregnancy (15). A recent nationwide prospective French cohort reported that 54% of primiparous women with chronic hypertension before pregnancy were treated with an antihypertensive agent during pregnancy; although the specific agents were not provided, the most commonly prescribed drug classes in this registry were beta blockers (52%) and calcium channel blockers (19%) (17). Together with our observation that labetalol and nifedipine are the two most commonly used agents in our HDP cohort, these findings collectively suggest that labetalol and nifedipine are likely to remain the most commonly used agents in the setting of gestational hypertension and preeclampsia as their use increases in the setting of chronic hypertension. These data illustrate the importance of rigorously evaluating gestational and post-partum changes in labetalol and nifedipine pharmacokinetics and response, and developing strategies to more precisely optimize the selection and dosing of these agents in individual patients (11).

This study has several limitations. Medication data was limited to prescriptions entered through the EHR. To have a complete analysis with reliable medication data, patients without medication data documented in the EHR were excluded, which could introduce selection bias and decrease generalizability. In addition, data on the specific doses prescribed, route of administration (intravenous versus oral), and whether medications or doses changed throughout the course of the pregnancy were not readily available, and we did not distinguish medication use in inpatient versus outpatient settings. If a patient's EHR documented two or more antihypertensive medications, we were unable to distinguish whether the patient was receiving concomitant therapy or whether medications were switched. We showed an association between antihypertensive therapy and several baseline factors, notably HDP severity; however, given the retrospective nature of this analysis, a causal inference cannot be made. In addition, documentation of the prescribers' rationale for the decision to treat or select a specific agent was not available, and thus we inferred that the observed relationships between demographic and clinical factors were directly considered in the medication ordering decision. Patients diagnosed with chronic hypertension in the absence of superimposed preeclampsia were not included in our cohort. Moreover, our results reflect prescribing patters at two academic medical centers in central North Carolina, and therefore may not be generalizable to other populations and clinical settings where healthcare systems and local guidelines may yield different pattern of antihypertensive medication use. Lastly, associations between medication use, BP changes, and clinical outcomes were not assessed, and we were unable to differentiate between deliveries due to lack of BP control despite medical treatment and deliveries for other reasons. Future studies in more geographically diverse populations can help advance our understanding of how antihypertensive medications are selected and dosed by prescribers to optimize outcomes in women with HDP.

In summary, the results of our study offer insight into the use and selection of antihypertensive medications during pregnancy in a large, diverse, real-world clinical setting. We observed that among patients diagnosed with HDP, antihypertensive medication use was common, and several demographic and clinical factors were associated with the decision to prescribe an antihypertensive. Specifically, HDP type and severity appeared to most strongly impact whether a patient received treatment with an antihypertensive medication. Our study also illustrated that beta blockers, specifically labetalol, was the most frequently ordered medication class in the treatment of each HDP type, while HDP type, known history of hypertension, and demographic factors were associated with the use of other medications such as nifedipine and hydralazine. Despite guideline recommendations, methyldopa was used very infrequently in clinical practice. Further clinical studies are needed to evaluate the use, safety, and effectiveness of antihypertensive medications in pregnant patients with distinct HDP diagnoses.

## Data Availability

The raw data supporting the conclusions of this article will be made available by the authors, without undue reservation.
