# Supplementary material for: Antihypertensive medication use during pregnancy in a real-world cohort of patients diagnosed with a hypertensive disorder of pregnancy
Source: Front Cardiovasc Med. 2023 Jul 7;10:1225251. doi: 10.3389/fcvm.2023.1225251 (PMC10360165; doi:10.3389/fcvm.2023.1225251)
Supplement: Supplementary file 1 [file Datasheet1.pdf]

Supplemental Figure 1. *Summary of the study population included in the analysis.* Flow diagram summarizing the inclusion and exclusion criteria used to identify the study population.

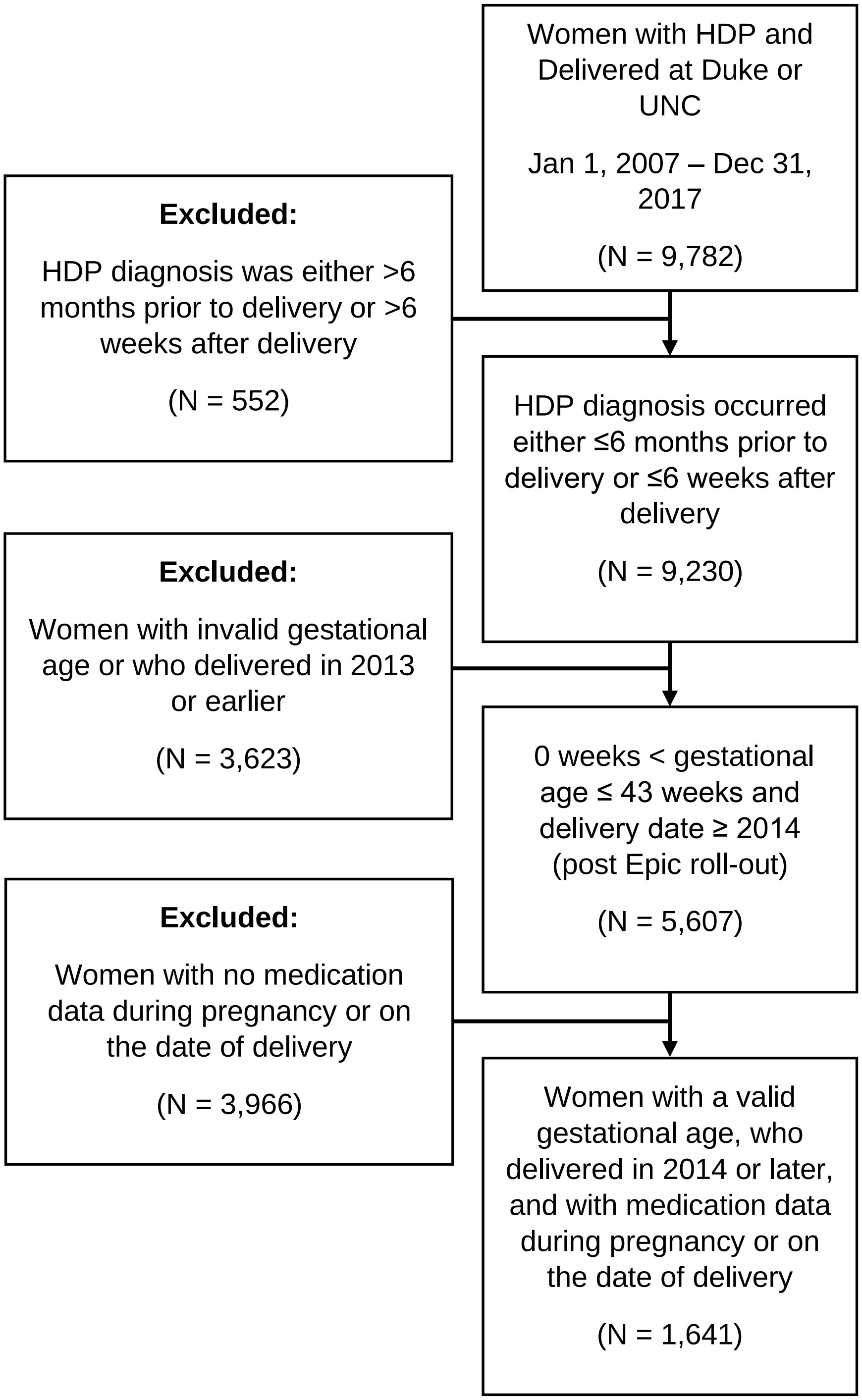

Supplemental Figure 2: *Sensitivity analysis of medication use in patients who delivered in 2016 and 2017*. (A) The proportion of patients who delivered in 2016-2017 (N=1079) that were treated with an antihypertensive agent stratified by HDP type. (B) The frequency of antihypertensive medication classes used among 2016-17 patients treated for HDP (N = 813) stratified by HDP type.

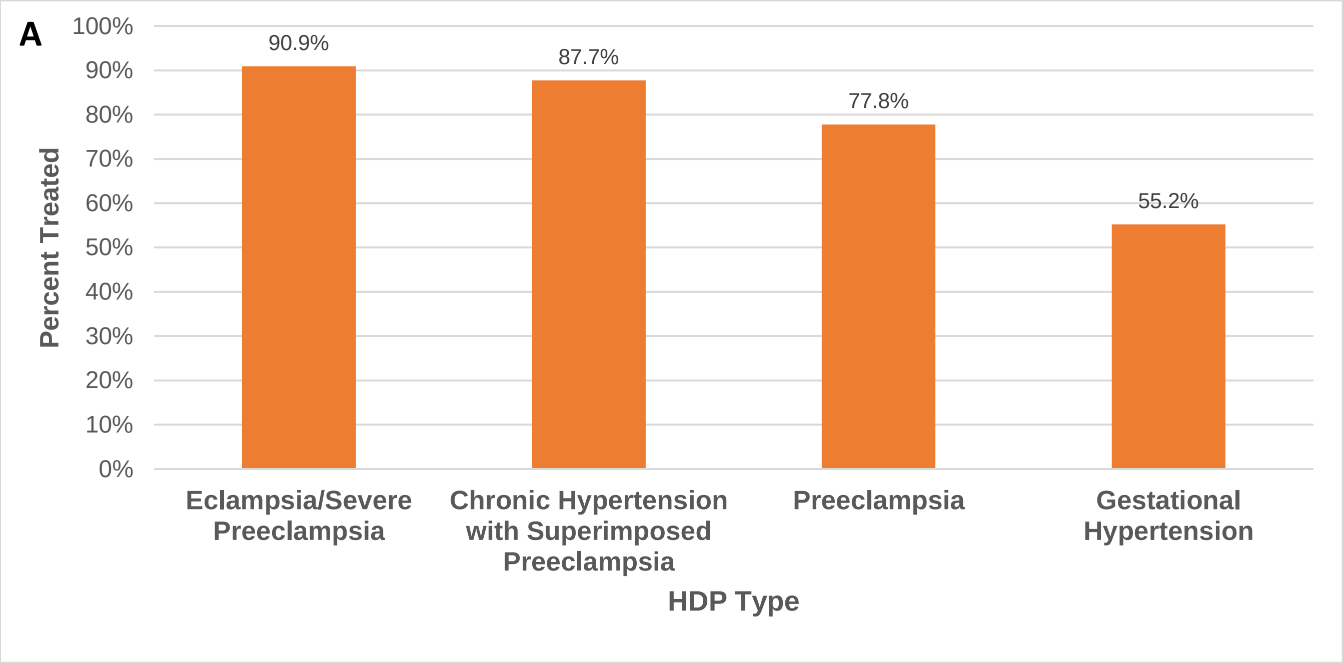

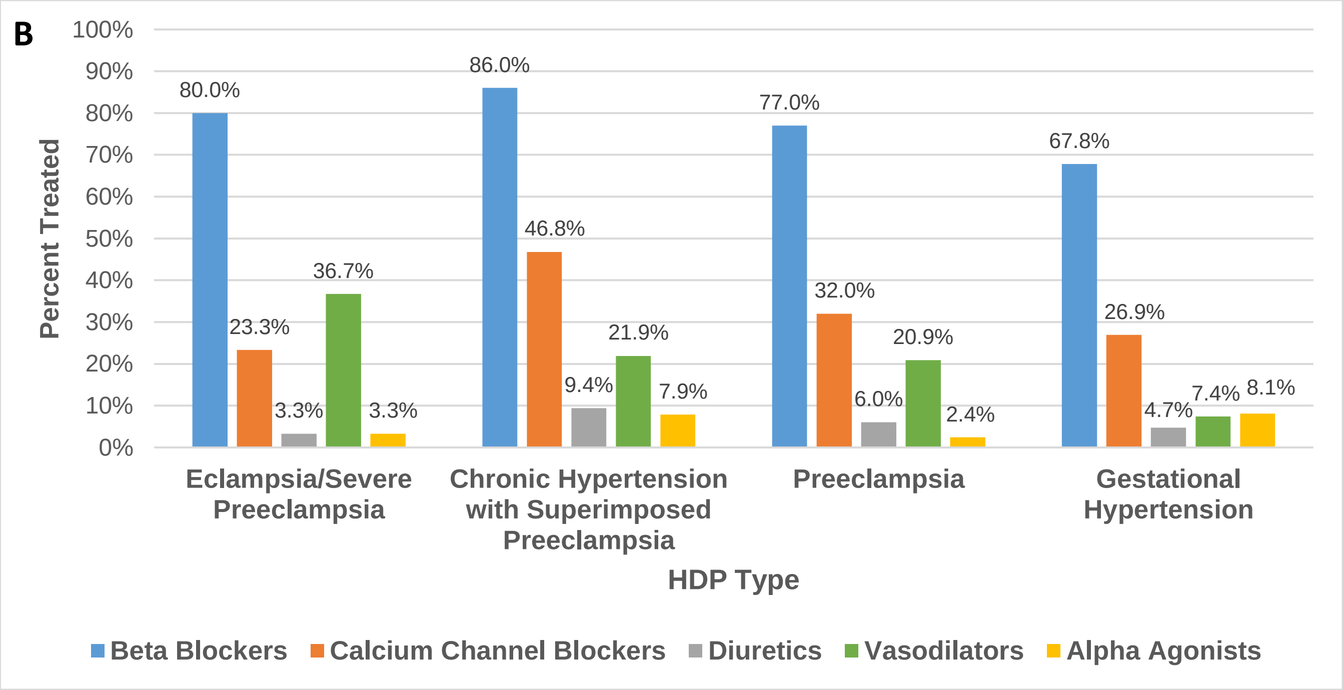

Supplemental Table 1: Demographic and clinical factors across treatment with a beta blocker (BB)

Demographic Characteristics	BB Yes (N = 1011)	BB No (N = 265)	Unadjusted OR	95% CI	P value	Adjusted OR ^	95% CI	P Value
**Age (years)**	30.5 ± 6.7	29.9 ± 6.3	1.02	0.99, 1.04	0.145	1.02	0.99, 1.05	0.145
**Black Race**	432 (42.7%)	102 (38.5%)	1.19	0.90, 1.57	0.213	1.16	0.81, 1.64	0.419
**Hispanic Ethnicity**	149 (14.7%)	36 (13.6%)	1.10	0.74, 1.63	0.635	1.25	0.74, 2.11	0.411
**Year of Delivery**								
2014-2015	374 (37.0%)	89 (33.6%)	*Reference*			*Reference*		
2016-2017	637 (63.0%)	176 (66.4%)	0.86	0.65, 1.15	0.304	0.69	0.44, 1.10	0.120
**Site of Delivery**								
Site A	451 (44.6%)	140 (52.8%)	*Reference*			*Reference*		
Site B	560 (55.4%)	125 (47.2%)	1.39	1.06, 1.82	0.017	1.31	0.94, 1.83	0.105
**Primary Payor**								
Government	513 (50.7%)	127 (47.9%)	*Reference*			*Reference*		
Commercial	394 (39.0%)	121 (45.7%)	0.81	0.61, 1.07	0.133	0.87	0.61, 1.24	0.437
Other (including self-pay)	104 (10.3%)	17 (6.4%)	1.51	0.88, 2.62	0.138	1.51	0.82, 2.76	0.186
**Hypertensive Disorders of Pregnancy (HDP)**								
Eclampsia/Severe preeclampsia	222 (22.0%)	38 (14.3%)	2.76	1.78, 4.28	<0.001	2.00	1.04, 3.82	0.037
Chronic hypertension with superimposed preeclampsia	299 (29.6%)	55 (20.8%)	2.57	1.73, 3.81	<0.001	2.44	1.50, 3.96	<0.001
Preeclampsia	329 (32.5%)	96 (36.2%)	1.62	1.13, 2.31	0.008	1.55	1.03, 2.34	0.035
Gestational hypertension	161 (15.9%)	76 (28.7%)	*Reference*			*Reference*		
**Pregnancy Characteristics**								
Gestational Onset of HDP (weeks)*	34.3 ± 4.6	34.8 ± 5.0	0.98	0.94, 1.01	0.179	1.00	0.96, 1.03	0.892
Gestational Diabetes	169 (16.7%)	50 (18.9%)	0.86	0.61, 1.22	0.409	0.70	0.45, 1.10	0.119
**Past Medical History**								
Hypertension	358 (35.4%)	73 (27.6%)	1.44	1.07, 1.94	0.016	1.16	0.79, 1.71	0.447
Type 1 DM	31 (3.1%)	7 (2.6%)	1.17	0.51, 2.68	0.718	0.55	0.19, 1.55	0.257
Type 2 DM	94 (9.3%)	16 (6.0%)	1.60	0.92, 2.76	0.095	1.66	0.83, 3.31	0.149
Prediabetes	42 (4.2%)	11 (4.2%)	1.00	0.51, 1.97	0.998	0.93	0.43, 2.03	0.861
Renal Disease	24 (2.4%)	3 (1.1%)	2.12	0.63, 7.11	0.222	4.86	0.61, 38.59	0.135

*Data only available in N = 1033. ^Adjusted analysis was conducted in the N = 1033 with gestational onset of HDP data available

Supplemental Table 2: Demographic and clinical factors across treatment with a calcium channel blocker (CCB)

Demographic Characteristics	CCB Yes (N = 406)	CCB No (N = 870)	Unadjusted OR	95% CI	P value	Adjusted OR ^	95% CI	P value
**Age**	30.9 ± 6.2	30.1 ± 6.8	1.02	1.00, 1.04	0.045	1.00	0.98, 1.03	0.684
**Black Race**	206 (50.7%)	328 (37.7%)	1.70	1.34, 2.16	<0.001	1.42	1.04, 1.94	0.029
**Hispanic Ethnicity**	47 (11.6%)	138 (15.9%)	0.69	0.49, 0.99	0.044	1.13	0.71, 1.81	0.609
**Year of Delivery**								
2014-2015	117 (28.8%)	346 (39.8%)	*Reference*			*Reference*		
2016-2017	289 (71.2%)	524 (60.2%)	1.63	1.27, 2.10	<0.001	2.45	1.58, 3.80	<0.001
**Site of Delivery**								
Site A	244 (60.1%)	347 (39.9%)	*Reference*			*Reference*		
Site B	162 (39.9%)	523 (60.1%)	0.44	0.35, 0.56	<0.001	0.41	0.30, 0.55	<0.001
**Primary Payor**								
Government	205 (50.5%)	435 (50.0%)	*Reference*			*Reference*		
Commercial	167 (41.1%)	348 (40.0%)	1.02	0.79, 1.31	0.886	1.01	0.73, 1.40	0.968
Other (including self-pay)	34 (8.4%)	87 (10.0%)	0.83	0.54, 1.27	0.393	1.01	0.61, 1.67	0.974
**Hypertensive Disorders of Pregnancy (HDP)**								
Eclampsia/Severe preeclampsia	57 (14.0%)	203 (23.3%)	0.74	0.49, 1.12	0.155	0.82	0.45, 1.52	0.532
Chronic hypertension with superimposed preeclampsia	158 (38.9%)	196 (22.5%)	2.13	1.50, 3.04	<0.001	1.63	1.05, 2.51	0.028
Preeclampsia	126 (31.0%)	299 (34.4%)	1.12	0.78, 1.59	0.546	0.95	0.63, 1.45	0.827
Gestational hypertension	65 (15.0%)	172 (19.8%)	*Reference*			*Reference*		
**Pregnancy Characteristics**								
Gestational Onset of HDP (weeks)*	33.3 ± 5.0	35.0 ± 4.4	0.93	0.90, 0.96	<0.001	0.92	0.89, 0.95	<0.001
Gestational Diabetes	86 (21.2%)	133 (15.3%)	1.49	1.10, 2.01	0.010	1.20	0.80, 1.81	0.382
**Past Medical History**								
Hypertension	164 (40.4%)	267 (30.7%)	1.53	1.20, 1.96	<0.001	1.26	0.90, 1.75	0.180
Type 1 DM	21 (5.2%)	17 (2.0%)	2.74	1.43, 5.25	0.002	2.44	0.99, 6.02	0.052
Type 2 DM	45 (11.1%)	65 (7.5%)	1.54	1.03, 2.30	0.033	0.96	0.55, 1.66	0.880
Prediabetes	15 (3.7%)	38 (4.4%)	0.84	0.46, 1.55	0.575	0.70	0.33, 1.49	0.354
Renal Disease	20 (4.9%)	7 (0.80%)	6.39	2.68, 15.23	<0.001	5.84	1.96, 17.39	0.002

*Data only available in N = 1033. ^Adjusted analysis was conducted in the N = 1033 with gestational onset of HDP data available

Supplemental Table 3: Demographic and clinical factors across treatment with a vasodilator (VD)

**Demographic Characteristics**	**VD Yes (N = 275)**	**VD No (N = 1001)**	**Unadjusted OR**	**95% CI**	**P value**	**Adjusted OR ^**	**95% CI**	**P value**
**Age**	30.3 ± 6.6	30.4 ± 6.6	1.00	0.98, 1.02	0.799	1.01	0.98, 1.04	0.446
**Black Race**	139 (50.6%)	395 (39.5%)	1.57	1.20, 2.05	0.001	1.63	1.13, 2.34	0.008
**Hispanic Ethnicity**	42 (15.3%)	143 (14.3%)	1.08	0.74, 1.57	0.681	1.36	0.82, 2.26	0.234
**Year of Delivery**								
2014-2015	118 (42.9%)	345 (34.5%)	*Reference*			*Reference*		
2016-2017	157 (57.1%)	656 (65.5%)	0.70	0.53, 0.92	0.010	1.12	0.70, 1.78	0.639
**Site of Delivery**								
Site A	114 (41.5%)	477 (47.7%)	*Reference*			*Reference*		
Site B	161 (58.6%)	524 (52.4%)	1.29	0.98, 1.68	0.068	1.60	1.13, 2.27	0.009
**Primary Payor**								
Government	148 (53.8%)	492 (49.2%)	*Reference*			*Reference*		
Commercial	94 (34.2%)	421 (42.1%)	0.74	0.56, 0.99	0.044	0.92	0.63, 1.34	0.647
Other (including self-pay)	33 (12.0%)	88 (8.8%)	1.25	0.80, 1.94	0.326	1.48	0.88, 2.46	0.137
**Hypertensive Disorders of Pregnancy (HDP)**								
Eclampsia/Severe preeclampsia	88 (32.0%)	172 (17.2%)	5.87	3.44, 10.02	<0.001	5.70	2.86, 11.34	<0.001
Chronic hypertension with superimposed preeclampsia	83 (30.2%)	271 (27.1%)	3.51	2.07, 5.97	<0.001	2.72	1.47, 5.01	0.001
Preeclampsia	85 (30.9%)	340 (34.0%)	2.87	1.70, 4.85	<0.001	2.73	1.51, 4.93	0.001
Gestational hypertension	19 (6.9%)	218 (21.8%)	*Reference*			*Reference*		
**Pregnancy Characteristics**								
Gestational Onset of HDP (weeks)*	33.3 ± 5.0	35.0 ± 4.4	0.91	0.88, 0.94	<0.001	0.92	0.89, 0.95	<0.001
Gestational Diabetes	46 (16.7%)	173 (17.3%)	0.96	0.67, 1.37	0.829	0.90	0.55, 1.46	0.664
**Past Medical History**								
Hypertension	94 (34.2%)	337 (33.7%)	1.02	0.77, 1.36	0.873	0.95	0.65, 1.39	0.802
Type 1 DM	11 (4.0%)	27 (2.7%)	1.50	0.74, 3.07	0.263	1.45	0.56, 3.73	0.446
Type 2 DM	24 (8.7%)	86 (8.6%)	1.02	0.63, 1.63	0.943	0.88	0.47, 1.66	0.701
Prediabetes	9 (3.3%)	44 (4.4%)	0.74	0.35, 1.53	0.410	0.54	0.22, 1.32	0.176
Renal Disease	9 (3.3%)	18 (1.8%)	1.85	0.82, 4.16	0.138	1.41	0.54, 3.65	0.481

*Data only available in N = 1033. ^Adjusted analysis was conducted in the N = 1033 with gestational onset of HDP data available
